# Comparison of anti-Vi IgG responses between two clinical studies of typhoid Vi conjugate vaccines (Vi-DT vs Vi-TT)

**DOI:** 10.1371/journal.pntd.0008171

**Published:** 2020-03-23

**Authors:** Eun Young Lee, Ju Yeon Park, Deok Ryun Kim, Manki Song, Sushant Sahastrabuddhe, Hun Kim, Yun Chon, Jae Seung Yang

**Affiliations:** 1 Clinical Research Laboratory, International Vaccine Institute, Seoul, Republic of Korea; 2 Biostatistics & Data Management, International Vaccine Institute, Seoul, Republic of Korea; 3 Development and Delivery, International Vaccine Institute, Seoul, Republic of Korea; 4 SK Bioscience, Seongnam, Republic of Korea; 5 Amgen Inc., Thousand Oaks, California, United States of America; The University of Sheffield, UNITED KINGDOM

## Abstract

*Salmonella enterica* serovar Typhi (*S*. Typhi) is a causative agent for typhoid fever and especially critical in developing countries. Although clinical studies for various typhoid conjugate vaccines (TCVs) have been performed, there are no comparative data on the immune responses of vaccines due to lack of harmonization of the serological assay. Recently, Typbar-TCV (Vi-TT) was prequalified by WHO and recommended for vaccination in endemic areas. Forty-eight serum samples were selected from a recent Vi-DT phase 1 study based on age cohort and anti-Vi IgG levels using an in-house ELISA. Anti-Vi IgG titers of 48 sera were also determined by Vacczyme ELISA, used in a Vi-TT phase 3 trial. A good correlation between the two assays was observed when the anti-Vi IgG titer was determined using Vacczyme ELISA based on the Vi-IgG_R1,2011_, U.S. reference reagent (Pearson correlation coefficient (*r*) = 0.991, *P* < 0.001) or Vacczyme ELISA calibrator (*r* = 0.991, *P* < 0.001). Based on the correlation, multiple linear regression model was developed to convert data of 281 sera (prior to vaccination and 28 days post first-dose) in the Vi-DT phase 1 study from in-house ELISA titers to Vacczyme ELISA values and then, compared with the Vi-TT results. Similar estimates of anti-Vi IgG GMT were observed after vaccination with the Vi-DT and Vi-TT vaccines [1626 EU/ml (95% CI: 1292–2047) vs 1293 EU/ml (95% CI: 1153–1449), respectively]. The method used here can be implemented to estimate and compare anti-Vi IgG levels between different clinical studies of TCVs. This approach enables comparison of the antibody responses among TCVs under development and may help facilitate licensing of new TCVs.

## Author summary

Typhoid fever is an infectious and life-threatening disease in developing countries. Before 2017, Ty21a and Vi polysaccharide vaccines were licensed but these are not recommended in young children under 2-year-old. Vaccine manufacturers are developing typhoid Vi conjugate vaccines (TCVs) to improve immunogenicity. Typbar-TCV (Vi-TT) demonstrated its safety and immunogenicity in infants and recently, prequalified by WHO. Serum Vi-specific IgG antibody has been used to measure the immunogenicity of TCV in many clinical trials. However, due to lack of harmonized assay, comparison of the immunogenicity among various TCVs is not possible in the absence of head-to-head clinical trials. Recently, we evaluated immunogenicity of Vi-DT using an in-house ELISA in the clinical study. In this study, 48 sera were selected from Vi-DT phase 1 study and measured anti-Vi IgG using commercial Vacczyme ELISA kit, used in the phase 3 study of Vi-TT, to compare the immunogenicity between two vaccines. Based on the correlation between two assays, anti-Vi IgG of all participants in the Vi-DT study was converted to Vacczyme antibody value using statistical model and compared with results of Vi-TT phase 3 study. The antibody levels induced by two studies were similar in pre- and post-vaccinated sera. This approach enables to compare the antibody responses among TCVs under development and would facilitate licensing of new TCVs.

## Introduction

Typhoid fever is a major global public health problem, especially in developing countries in South and South-East Asia and sub-Saharan Africa. *Salmonella enterica* serovar Typhi (*S*. Typhi) is the causative pathogen of typhoid fever, a disease that is potentially life-threatening without proper and timely treatment. The burden of typhoid fever has been estimated to be 26.9 million cases and 216,000 deaths per year [1, 2]. Infants and children under 15 years of age are more vulnerable to infection (~81%) than the overall population [3]. Since the disease is transmitted via contaminated water and food, improvements to sanitation and hygiene in endemic regions represent the ultimate prevention strategy but are costly and time-consuming. In the interim, vaccination is the most cost-effective approach for reducing the burden of typhoid fever in vulnerable communities.

Before 2017, two typhoid vaccines were available globally to prevent typhoid fever. The first is the live-attenuated *S*. Typhi Ty21a oral vaccine. This mutant strain is highly attenuated by chemical inactivation and lacks the galactose-epimerase (*galE*) gene and Vi capsule polysaccharide [4, 5]. It results in 67–80% protective efficacy and immune responses sustained for up to 7 years after vaccination [4, 6]. The other vaccine is injectable Vi polysaccharide, exhibiting over 70% efficacy in highly endemic areas and herd protection in community trials [7, 8]. Of these two vaccines, the plain Vi vaccine was prequalified by the WHO in 2011 and is available for acquisition by Gavi/UNICEF to support public health programs. However, neither vaccine is recommended for use in children under 2 years of age due to capsule availability of oral vaccine and poor immunogenicity of plain Vi vaccine [9, 10]. To improve the immunogenicity of the Vi vaccine, especially in young children under age 2, typhoid conjugate vaccines (TCVs) are being developed, in which Vi is conjugated to a non-toxic carrier protein, including recombinant exoprotein A from *Pseudomonas aeruginosa* (Vi-rEPA), tetanus toxoid (Vi-TT), cross-reacting material (Vi-CRM_197_), and diphtheria toxoid (Vi-DT) [11]. The efficacy trial of the Vi-rEPA vaccine showed that the Vi conjugate protected children aged 2 to 5 years in a two-dose regimen at 90% efficacy for 4 years and anti-Vi IgG persisted for 10 years [11, 12]. Clinical studies of the Vi-TT vaccine (Typbar-TCV from Bharat Biotech, India) demonstrated it to be safe and highly immunogenic in children aged 6 months to 2 years, with a 98% seroconversion rate [13]. Recently, Typbar-TCV was prequalified by the WHO, followed by a WHO recommendation to introduce TCVs in children over 6 months of age in endemic countries. There are two additional Vi-TT vaccines (Pedatyph^TM^, Biomed, India; Zydus Cadila, India) licensed in India [14] and other TCVs in various clinical trial phases globally.

Vi is known to be a major protective antigen against *S*. Typhi [15]. Enzyme-linked immunosorbent assay (ELISA) has been widely used to quantify anti-Vi IgG levels in serum, with protective serum anti-Vi IgG levels estimated at 3.52 ELISA units/ml, equivalent to 4.3 μg/ml [12] in children aged 2–5 years in a passive surveillance study [16]. Since then, a number of clinical studies have evaluated the safety and immunogenicity of other TCVs, however protective antibody levels were not defined and could not be compared due to the lack of comparative ELISA studies and the absence of a validated reference serum, which was not available at the time.

The National Institute for Biological Standards and Control (NIBSC, UK) led a WHO collaborative study with 10 different laboratories from 7 countries to evaluate the potency of both the Vi-IgG_R1,2011_ and the candidate human international standard (IS), NIBSC 10/126, for determining human anti-typhoid Vi IgG levels using the commercial Vacczyme ELISA and participants’ in-house ELISAs. The results showed that the relative potency of the candidate IS compared to Vi-IgG_R1, 2011_ was consistent in the Vacczyme ELISA, but high variability within the range of 0.20 to 6.24 for the potency of IS 10/126 was observed depending on the ELISA format [17]. Following this study, the NIBSC organized an additional WHO collaborative study with 7 research groups from 6 countries to clarify the variation among in-house ELISAs and to establish the first human IS, NIBSC 16/138, due to the paucity of Vi-IgG_R1, 2011_ and NIBSC 10/126. In the latter study, the anti-Vi IgG concentrations of 6 serum samples and Vi IgG _R1, 2011_ were determined based on NIBSC 16/138 using in-house ELISAs and the Vacczyme ELISA. NIBSC 16/138, NIBSC 10/126, and Vi-IgG_R1, 2011_ were assigned values of 100 international units (IU), 54 IU, and 163 IU per ampoule, respectively [18]. However, only 3 laboratories’ methods, including our in-house assay, showed overall potencies of anti-Vi IgG consistent with the Vacczyme. In particular, it was reported that our assay (designated as in-house ELISA 1) could be a suitable non-commercial alternative to the Vacczyme because it had been established successfully in another laboratory [18].

Recently, in a collaborative study between the International Vaccine Institute (IVI) and SK Biosciences (South Korea), we demonstrated that the Vi-DT conjugate vaccine is safe and highly immunogenic in healthy Filipino children and adults in a phase 1 study [19]. In this study, the anti-Vi IgG responses to the Vi-DT vaccine in sera were measured by our in-house ELISA [19], whereas in a phase 3 study the responses to Vi-TT were assessed by the commercially available Vacczyme ELISA [13]. To determine the comparability between the Vacczyme ELISA and our in-house ELISA, the anti-Vi IgG titers were measured in both assays using 48 samples from Vi-DT phase 1 study and their correlation was assessed. Based on this correlation, the predicted Vacczyme ELISA values for the anti-Vi IgG levels of all 281 sera from the Vi-DT phase 1 study were used to compare the immune responses between the clinical trial studies of Vi-TT and Vi-DT [13, 19].

## Methods

### Ethics statement

Use of serum samples was approved by the institutional review boards both of RITM (2015-38-1) and IVI (2015–005). Written informed consent was received from each study participant.

### Serum samples

Seventy-two healthy Filipino adults and children participated in the phase 1 clinical trial of the Vi-DT conjugate vaccine at the Research Institute for Tropical Medicine (RITM) in Manila, the Philippines (Clinicaltrials.gov: NCT02645032) in the previous study [19]. Participants were randomized to receive Vi-DT or Typhim Vi (plain Vi) within three age cohorts, adults (18–45 years of age), adolescents (6–17 years of age), and children (2–5 years of age), as described previously [19]. A total of 216 serum samples obtained from participants vaccinated with Vi-DT in the phase 1 clinical trial were assessed to determine anti-Vi IgG levels using in-house Vi-ELISA [19], and 48 samples among these were selected for Vacczyme ELISA based on serum anti-Vi IgG levels within each age cohort. Samples from participants who had elevated liver function tests or other vaccine history were excluded to avoid potential confounding factors for elevated IgG levels. Exceptionally low (undetectable) and high (>95%) values were also excluded to avoid effect of extreme values in the prediction model. Measured serum anti-Vi IgG levels were categorized into low, medium, and high using cut-offs of 29.1% and 66.7%. The number of samples in each group in each age cohort are shown in Table 1. The U.S. reference reagent, Vi IgG_R1, 2011_, was kindly provided by Dr. Shousun C. Szu at the National Institutes of Health, USA. It has been assigned an anti-Vi IgG titer of 33 μg/ml [12] and was used to determine anti-Vi IgG levels in test sera as a reference serum for in-house ELISA.

**Table 1:** Details on the 48 samples selected from the Vi-DT phase 1 study for Vacczyme ELISA.

Anti-Vi IgG Age group	Low	Medium	High	Total
Adults (18–45 yrs)	5	5	5	15
Adolescents (6–17 yrs)	4	6	6	16
Children (2–5 yrs)	5	7	5	17
Total	14	18	16	**48**

* Anti-Vi IgG (μg/ml) < 20.279 (Low); ≥20.279 and <51.143 (Medium); ≥51.143 (High).

### In-house Vi-ELISA

In-house assay was used to measure anti-Vi-specific IgG antibodies in human sera. Briefly, 96-well plates were precoated with 10 μg/ml poly-l-lysine (Sigma-Aldrich, USA). After washing the plates, 2 μg/ml Vi antigen (SK Bioscience, South Korea) was absorbed overnight at 37°C. After blocking with 1% bovine serum albumin in PBS, diluted human sera and reference serum were added to the plate and incubated for 1 h at 37°C. Alkaline phosphatase (AP)-conjugated mouse anti-human IgG (Abcam, UK) was added and incubated for 1 h. After washing, 4-nitrophenyl phosphate substrate (Sigma-Aldrich) was added for 1 h. The plate was read at 405 nm and corrected at 490 nm. Anti-Vi IgG titer was calculated based on U.S. reference serum Vi IgG_R1, 2011_.

### Commercially available Vi-ELISA (Vacczyme ELISA)

Forty-eight selected samples were assessed using the Vacczyme human anti-*S*. Typhi Vi IgG enzyme immunoassay kit (Binding site, UK) according to the manufacturer’s instructions. The anti-Vi IgG titers of serum samples including Vi IgG_R1, 2011_ were examined and calculated using either the Vacczyme ELISA calibrator or Vi IgG_R1, 2011_.

### Statistical analysis

The geometric mean (GM) and geometric standard deviation (SD) of the anti-Vi IgG titers are presented overall and by age cohort. The correlation coefficient between assays was calculated on a log-transformed scale overall and by age cohort. A scatter plot and linear mean regression line with 95% confidence interval were presented. To assess agreement between assays, a Bland–Altman plot was constructed from standardized data, after subtracting the mean and dividing by the standard deviation, owing to differences in units between the two assays.

To establish the prediction model, the association between the in-house ELISA and Vacczyme (calibrator) was assessed using a multiple linear regression model of assay values obtained from the Vacczyme (calibrator) (*Y*_*i*_) and in-house ELISA (*X*_1*i*_) values as:

$$Y_{i}={\hat{\beta }}_{0}+{\hat{\beta }}_{1}X_{1i}+{\hat{\beta }}_{2}X_{2i}+{\hat{\beta }}_{3}X_{3i}+{\hat{\beta }}_{4}X_{4i}+{\hat{\beta }}_{5}X_{5i}+e_{i},$$

where the errors are assumed to be independent, identical, and normally distributed with mean 0 and variance σ^2^. Covariate variables (*X*_2*i*_, *X*_3*i*_) are dummies for age group (adults and adolescents, respectively). In-house standard by age cohort interactions were also included in the prediction model as *X*_4*i*_ and *X*_5*i*_ for adults and adolescents, respectively. Log-transformed dependent and independent variables were used to fit the linear regression model. To predict the Vacczyme ELISA value of the anti-Vi IgG titer (EU/ml) in the Vi-DT phase 1 study, the model was fitted (R^2^ = 0.99), and estimates (βs) of the linear regression model were obtained using data from 48 samples. A total of 1,030 sets of 148 predicted values of ${\hat{Y}}_{ib}$ from a fixed- log scale of X samples (*X*_1*i*_, *X*_2*i*_, *X*_3*i*_, *X*_4*i*_, *X*_5*i*_) from Vi-DT phase 1 study individuals and samples of *e*_*ib*_ ~ (0,σ^2^) were generated to consider the prediction error for each in-house Vi-ELISA value. For each set *b*, we calculated the average $\hat{Y_{b}}$ over 148 individual values of *Y*_*ib*_ and the confidence interval of $\hat{Y_{b}}$. The predicted value of the geometric mean was calculated as the exponential of average of ${\hat{Y}}_{b}$ over 1,030 sets, and the predicted interval was calculated as the exponential of average of the confidence interval of $\hat{Y_{b}}$.

## Results

### Comparison between the in-house Vi-ELISA and the Vacczyme ELISA

**Fig 1:**
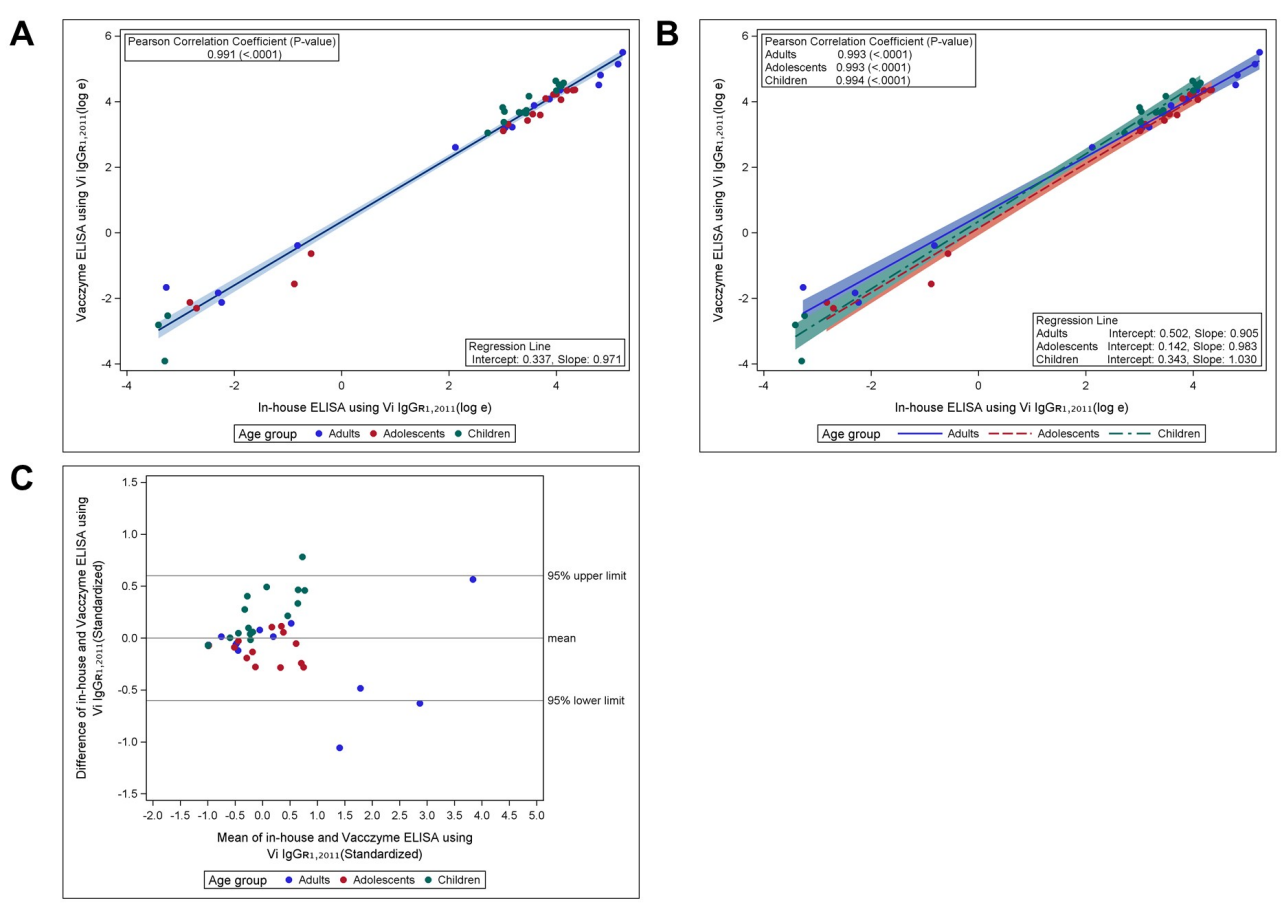
Correlation of serum anti-Vi IgG values determined with U.S. reference reagent Vi IgG_R1, 2011_ between in-house Vi-ELISA and Vacczyme ELISA. Scatter plot and linear mean regression line with 95% confidence interval (A) overall and (B) by age group. Correlations between the assays were analyzed using Pearson correlation coefficient (*P* < 0.001). (C) Correlation between the assays according to Bland–Altman plot using standardized data.

**Fig 2:**
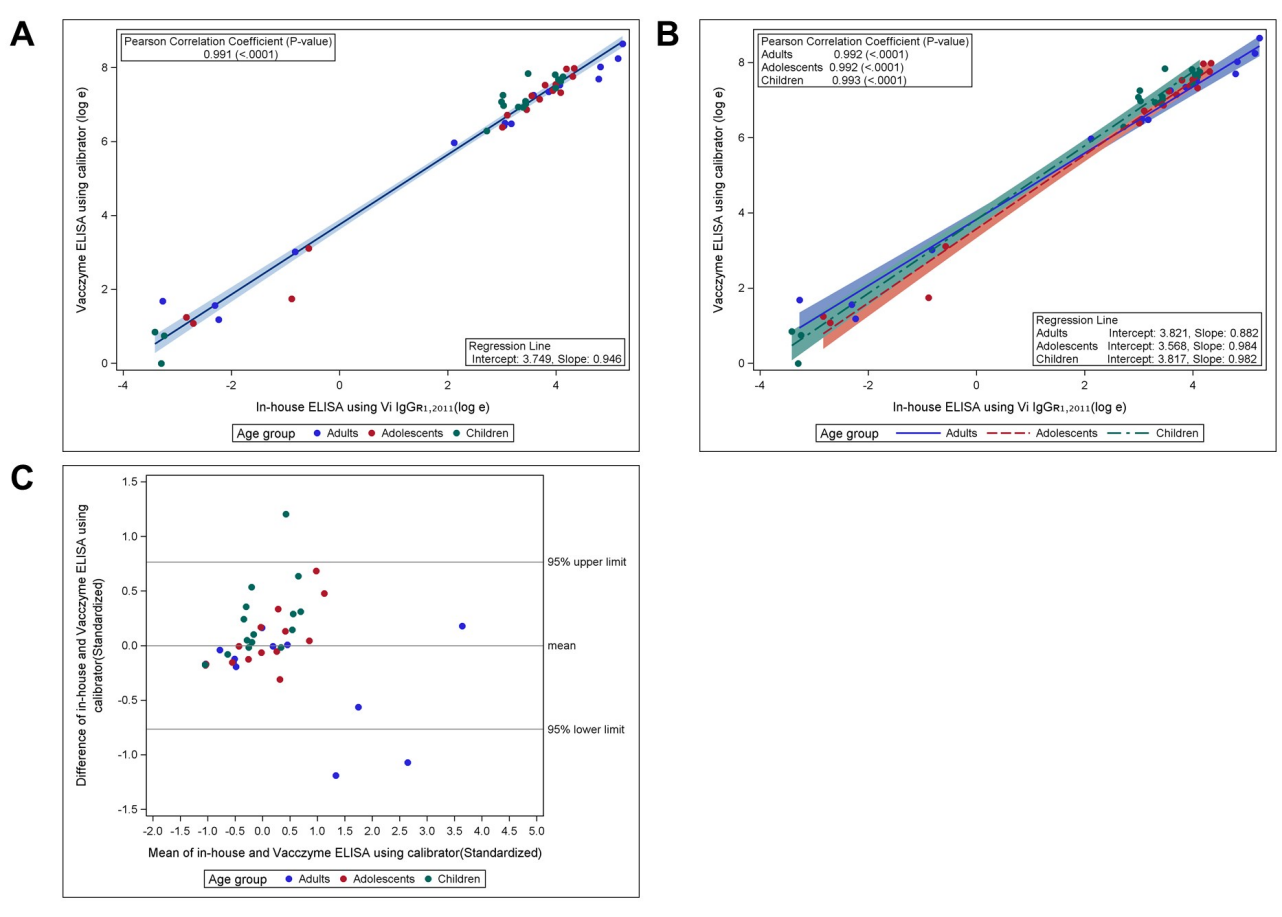
Correlation of serum anti-Vi IgG values between in-house Vi-ELISA using Vi IgG_R1, 2011_ and Vacczyme ELISA using calibrator. Scatter plot and linear mean regression line with 95% confidence interval (A) overall and (B) by age group. Correlations between the assays were analyzed using Pearson correlation coefficient (*P* < 0.001). (C) Correlation between the assays according to Bland–Altman plot using standardized data.

To assess the comparability between the in-house Vi-ELISA and Vacczyme ELISA, the anti-Vi IgG levels of 48 samples were measured using the Vacczyme ELISA kit, and antibody titers were calculated using either the U.S. reference reagent Vi IgG_R1, 2011_ or the kit’s calibrator as standard serum (Table 2). The overall GM titers (GMTs) of anti-Vi IgG for all 48 sera were 10.293 μg/ml (range: 0.033–187.588) using in-house ELISA and 13.469 μg/ml (range: 0.020–246.35) using the Vacczyme ELISA were calculated based on the Vi IgG_R1, 2011_ reference. The GMT of anti-Vi IgG among all samples was 385.492 EU/ml (range: 0.992–5694.64) based on the Vacczyme ELISA calibrator. Serum anti-Vi IgG levels determined by Vi IgG_R1, 2011_ showed a good correlation between the two ELISAs: the Pearson correlation coefficient between the two methods was *r* = 0.991 (*P* < 0.001), intercept = 0.337, slope = 0.971 in all age groups (Fig 1A), with values of *r* = 0.993 (*P* < 0.001) for adults, *r* = 0.993 (*P* < 0.001) for adolescents, and *r* = 0.994 (*P* < 0.001) for children (Fig 1B). When using Vi IgG_R1, 2011_ as a standard, the Bland–Altman plot of standardized data showed that differences in values between the in-house and Vacczyme ELISA were randomly scattered around the mean difference line without any pattern. Aside from three points, all differences were within the 95% upper and lower confidence limits (1.96SD), where the estimate of the SD of the difference was relatively small (1.96SD is less than 1 while, the SD of each assay value was fixed at 1 by standardization) (Fig 1C). In addition, the correlation between the two assays was demonstrated by determining anti-Vi IgG levels with the Vacczyme ELISA calibrator, resulting in Pearson correlation coefficients of *r* = 0.991 (*P* < 0.001), intercept = 3.749, slope = 0.946 for all age groups (Fig 2A), *r* = 0.992 (*P* < 0.001) for adults, *r* = 0.992 (*P* < 0.001) for adolescents, and *r* = 0.993 (*P* < 0.001) for children (Fig 2B). The Bland–Altman plot of Vacczyme-calibrated values was very similar to that of the Vi IgG_R1, 2011_-calibrated values (Fig 2C).

**Table 2:** Anti-Vi IgG values determined by in-house ELISA and Vacczyme ELISA.

Assays (Reference serum, Unit)	GMT ± SD	Median	Min	Max	CV
**In-house ELISA (Vi IgG**_**R1,2011,**_ μ**g/ml), n = 48**	10.293 ± 14.574	31.107	0.033	187.588	1.042
Adults, n = 15	9.831 ± 18.906	23.931	0.038	187.588	1.177
Adolescents, n = 16	11.098 ± 12.948	37.667	0.059	76.747	0.744
Children, n = 17	9.986 ± 15.099	29.709	0.033	62.335	0.691
**Vacczyme ELISA (Vi IgG**_**R1,2011,**_ μ**g/ml), n = 48**	13.469 ± 13.807	39.430	0.020	246.350	0.971
Adults, n = 15	13.080 ± 14.582	25.540	0.120	246.350	1.191
Adolescents, n = 16	12.276 ± 12.607	36.875	0.100	78.310	0.746
Children, n = 17	15.082 ± 16.670	40.450	0.020	102.800	0.704
**Vacczyme ELISA (Calibrator, EU/ml), n = 48**	385.492 ± 12.927	1191.135	0.992	5694.640	0.881
Adults, n = 15	342.311 ± 13.614	662.525	3.258	5694.640	1.131
Adolescents, n = 16	378.705 ± 12.676	1326.449	2.937	2922.788	0.784
Children, n = 17	435.313 ± 14.631	1184.335	0.992	2534.722	0.655

### Predictive value of anti-Vi IgG titers of Vi-DT conjugate vaccine

Because anti-Vi IgG responses are proven to be highly correlated between the two assays, antibody titers determined by one assay (e.g., in-house ELISA) should be highly predictive of antibody titers determined by the other assay (e.g., Vacczyme ELISA). Therefore, we used the multiple linear regression model fitted to data from the selected 48 samples to convert the anti-Vi IgG titers in μg/ml of all samples at day 0 and day 28 (n = 281; [19]) in the Vi-DT phase 1 study including plain Vi and Vi-DT groups into Vacczyme ELISA values in EU/ml (Table 3).

**Table 3:** Comparison of transformed serum anti-Vi IgG values from participants of the phase 1 study of Vi-DT and the phase 3 study of Vi-TT by Vacczyme ELISA.

Vi-DT P1*		All ages*		Adults (18–45 yrs)*		Adolescents (6–17 yrs)*		Children (2–5 yrs)*	
Response	Time point	Vi-DT	Vi	Vi-DT	Vi	Vi-DT	Vi	Vi-DT	Vi
Number of participants	Day 0	71	72	24	24	24	24	23	24
	Day 28	69	69	22	21	24	24	23	24
GMT EU/ml (95% prediction interval)	Day 0	3.3 (2.4, 4.6)	3.5 (2.5, 4.9)	10.4 (5.8, 18.5)	9.6 (5.3, 17.3)	2.0 (1.3, 3.1)	1.6 (1.1, 2.2)	1.7 (1.3, 2.3)	2.8 (1.7, 4.7)
	Day 28	1626 (1292, 2047)	402 (319, 508)	1575 (896, 2769)	300 (192, 469)	1636 (1089, 2458)	455 (319, 650)	1666 (1316, 2109)	460 (296, 715)
Vi-TT P3**		All ages**		Adults (16–45 yrs)**		Adolescents (5–15 yrs)**		Children (2–4 yrs)**	
Response	Time point	Vi-TT	Vi	Vi-TT	Vi	Vi-TT	Vi	Vi-TT	Vi
Number of participants	Day 0 & Day42	332	305	86	89	146	126	100	90
GMT EU/ml (95% confidence interval)	Day 0	10.4 (9.6, 11.3)	11.6 (10.5, 12.9	13.3 (11, 16)	14 (11, 17)	10.2 (9.1, 11.33)	11.1 (9.5, 12.9)	8.8 (8.0, 9.6)	10.0 (8.5, 11.7)
	Day 42	1293 (1153, 1449)	411 (359, 471)	781 (610, 1001)	378 (283, 504)	1701 (1473, 1965)	409 (334, 499)	1334 (1081, 1648)	454 (356, 578)

* Transformed Vacczyme ELISA GMT values of anti-Vi IgG pre- and post-vaccination in a randomized phase 1 study of Vi-DT vs Vi Polysaccharide (Typhim Vi, Sanofi Pasteur) using a multiple regression model. Vi-DT P1 denotes phase 1 study of Vi-DT.

** GMT value of anti-Vi IgG pre- and post-vaccination in a randomized phase 3 study of Vi-TT (Typbar-TCV) vs Vi Polysaccharide (Typbar, Bharat Biotech.) using Vacczyme ELISA [13]. Vi-TT P3 denotes phase 3 study of Vi-TT vaccine.

The overall GMTs of anti-Vi IgG (EU/ml) at day 0 in Vi-DT phase 1 study [19] were 3.3 (95% CI: 2.4–4.6) and 3.5 (95% CI: 2.5–4.9) in the Vi-DT and plain Vi vaccination groups, respectively (Table 3) while the overall GMTs were 10.4 (95% CI: 9.6–11.3) and 11.6 (95% CI: 10.5–12.9) in the Vi-TT and plain Vi vaccination groups at day 0 in the phase 3 study of the Vi-TT vaccine [13]. Notably, the antibody GMTs at day 28 after primary immunization in the Vi-DT study were comparable to those at day 42 in the Vi-TT study, with values of 1626 (95% CI: 1292–2047) and 1293 (95% CI: 1153–1449), respectively. Similarity was also observed between two plain Vi vaccines (Typhim Vi and Typbar), with values of 402 (95% CI: 319–508) and 411 (95% CI: 359–471) after vaccination, respectively.

## Discussion

The immunogenicity of Vi-based typhoid vaccines, including plain Vi vaccine and TCV, have been evaluated by measurements of anti-Vi IgG levels in serum using ELISA. Although anti-Vi IgG antibody levels have been shown to highly correlate with protection against typhoid fever, there is no known robust threshold level for clinical protection [10, 16, 20–22]. Typbar-TCV received WHO prequalification in December 2017, and a number of other candidates are undergoing clinical trials to obtain licensure. Despite several clinical studies of various TCVs to date, anti-Vi IgG responses elicited by various TCVs could not be compared due to the absence of a validated reference reagents for Vi and human anti-Vi serum [11, 13, 16, 19–23]. To address this issue, the WHO has organized a meeting to develop guidelines for the quality, safety, and efficacy of TCVs [24, 25].

Recently, the NIBSC established the first human IS, NIBSC 16/138, to determine anti-Vi IgG titers in serum [18] and it is currently available to assess anti-Vi IgG levels induced by various formulations of TCVs. Despite these efforts, a direct comparison of vaccine-induced antibody responses among TCVs is not straight forward due to the absence of head-to-head trials and the lack of a standardized assay, as observed previously [17]. Moreover, it is not feasible to compare results from the previous clinical trials when different assays are used to determine the immunogenicity of TCVs.

A good correlation between our in-house ELISA and Vacczyme ELISA was observed for anti-Vi IgG levels based on either US reference Vi IgG_R1, 2011_ or the Vacczyme ELISA’s calibrator in this study. This result supports the outcome of the previous collaborative study [18], which showed that our in-house ELISA and the Vacczyme determined a similar anti-Vi IgG potency for the US reference. Based on the correlation, we developed a statistical model to transform data from the in-house ELISA (μg/ml) to the Vacczyme ELISA (EU/ml) in the Vi-DT phase 1 study [19]. Interestingly, these values were highly comparable to GMT levels in post-vaccinated sera of the Vi-TT phase 3 study [13]. This finding is important because it is the first attempt to estimate and compare anti-Vi IgG levels between different clinical studies of TCVs. It potentially provides strong evidence that two TCVs could elicit similar antibody responses.

However, there are still some limitations in predicting Vacczyme ELSIA GMT values for comparison of the immunogenicity between the Vi-DT and Vi-TT vaccines. For instance, laboratory procedures, including serum dilution factors and the use of single or serial dilutions of serum for determination of anti-Vi IgG levels, between laboratories might affect the results, even though the anti-Vi IgG values of 48 samples from the Vi-DT phase 1 study were measured using Vacczyme ELISA to predict the Vacczyme ELISA GMT value. Additionally, given the fact that the baseline GMT in the Vi-TT phase 3 study was higher than that in the Vi-DT phase 1 (3.3 vs 10.4), the immunological characteristics of populations might differ between less-endemic and endemic areas. Finally, the predicted Vi-DT phase 1 data should be interpreted with caution, since these represent predicted values from the fitted model using a subset of data from the phase 1 study. Especially, transformed Vacczyme ELISA value of samples with low outliers in the Vi-DT phase 1 study were below limit of Vacczyme kit (< 7.4 EU/ml) when prediction model was applied. In addition, samples with high outliers may not be accurately predicted because the model was developed within the specific range of antibody although the variability caused by extreme outliers was incorporated in the estimate of error term and summary statistics of results for two studies. Therefore, it would be helpful and provide more accurate predictions of antibody concentrations if serum samples obtained from the two studies could be tested in both laboratories using our in-house ELISA and the Vacczyme ELISA kit.

In this study, we confirmed previous results of collaborative study that a good concordance was observed between the Vacczyme ELISA and the IVI in-house ELISA, and both assays are commutable [18]. Amongst the various ELISA formats used in this study, the IVI in-house ELISA was demonstrated as a credible non-commercial alternative for the Vacczyme ELISA. Especially, since the assay procedure was successfully transferred to another laboratory, and the results of both laboratories showed excellent parallelism and precision of the assay [18]. An extended study is currently in progress to evaluate our in-house ELISA more extensively through multi-nation collaboration. Thus, IVI in-house ELISA will be more reliable assay for clinical trials of TCVs if commutability of the assay is evident in the extended research.

In summary, this study showed that anti-Vi IgG responses are similar between the Vi-DT and Vi-TT vaccines based on predicted antibody GMT values. Although there is currently no standardized ELISA format across laboratories for predicting the immune responses of various TCVs, it may be feasible to compare anti-Vi IgG responses in cases in which there is a strong correlation and agreement between the two ELISA formats. Thus, the method used here enables comparison of the anti-Vi IgG results from different clinical studies, despite the limitations mentioned above, and may be helpful in facilitating the licensing of new typhoid vaccines.
